# Examining Body Fat Percentage, Galvanic Skin Response, and Muscle Grip Strength in Female Hypothyroid Patients

**DOI:** 10.7759/cureus.42023

**Published:** 2023-07-17

**Authors:** Prashanth A, Ruchi Kothari, Yogesh S, Gaurav Mittal, Shabbir Gheewala, Pradeep Bokariya, Amisha Palande, Devupriya M S, Shubhi Tamrakar, Sai Shanmukh Vemparala

**Affiliations:** 1 Physiology, Mahatma Gandhi Institute of Medical Sciences, Wardha, IND; 2 Research, Rotaract Club of Indian Medicos, Mumbai, IND; 3 Internal Medicine, Madras Medical College and Hospital, Rajiv Gandhi Government General Hospital, Chennai, IND; 4 Anatomy, Mahatma Gandhi Institute of Medical Sciences, Wardha, IND; 5 Physiology, Terna Medical College, Mumbai, IND; 6 Physiology, Dr. D. Y. Patil Medical College, Mumbai, IND; 7 Physiology, Andhra Medical College, Visakhapatnam, IND; 8 Physiology, Great Eastern Medical School and Hospital, Visakhapatnam, IND

**Keywords:** body mass index (bmi), anthropometry, hypothyroid, hand grip strength, body fat percentage, galvanic skin response

## Abstract

Background

The thyroid gland is an indispensable organ exerting control over the activity of multiple organ systems including the autonomic nervous system. This study attempted to monitor the variations in autonomic function parameters such as galvanic skin response (GSR) and muscle grip strength (HGS) in conjunction with changes in body fat percentage (BFP).

Methodology

This case-control study was conducted among 40 female hypothyroid patients as cases and 40 age-matched female healthy volunteers as controls. Anthropometric data were collected using standard techniques. GSR and HGS were measured using Equivital Sensory Electronic Module and Grip Force Transducer, respectively. Data extraction and analysis were done using the LabChart software.

Results

The mean age of the 40 female hypothyroid patients was 30.14 ± 5.91 years, whereas the mean age of the female controls was 29.37 ± 6.59 years. The waist circumference of cases was 85.81 ± 10.39 cm while that of controls was 80.90 ± 11.18 cm. The BFP of cases was 35.38% ± 6.74% while that of controls was 31.72% ± 5.63%. The GSR amplitude showed a significant difference between hypothyroid and healthy volunteers with values of 1.34 ± 1.14 μS and 2.40 ± 1.86 μS, respectively. The HGS indices showed no significant difference between the two groups. A statistically negative correlation was noted between BFP and GSR amplitude (-0.32), whereas a positive correlation was noted between BFP and mean handgrip strength (0.31) in hypothyroid patients.

Conclusions

The changes in BFP and autonomic function through GSR and HGS were evaluated in female hypothyroid patients with respect to healthy females. The interrelationship between anthropometry and autonomic function was also explored in this study. The findings of this study can augment prognosis in patients and ensure timely corrective treatment for improving quality of life.

## Introduction

Hormones are crucial for the regulation of cellular metabolic activities, some of which are also secreted by the thyroid gland. Hypothyroidism refers to a decrease in the level of these hormones, which can be due to primary or secondary causes. Hypothyroid patients can present with impairments, such as an increased prevalence of myocardial infarction, and a higher frequency of neuromuscular complaints, such as myalgia, weakness, and reduced muscle strength [1]. Among endocrine disorders, hypothyroidism is the second to diabetes mellitus in prevalence. In India, the prevalence of hypothyroidism is 10.95% [2,3]. Females are more affected by this disease, with postmenopausal and elderly females being the most vulnerable [3].

The sympathetic branch of the autonomic nervous system (ANS) innervates the human eccrine sweat glands, which are mostly located on the palmar surfaces of the hands, soles of the feet, and over the skin. In particular, the axilla and palm areas are connected not only to thermoregulation but also to emotional status. Therefore, it is possible to measure skin conductance, and the measured parameter is known as galvanic skin response (GSR). GSR is a parameter sensitive to the physiological changes in sweat gland activity on the skin which are non-conscious processes that are indicative of physiological or psychological arousal [4]. GSR is recorded to assess the sympathetic activity in an individual.

The thyroid hormone influences the ANS to a considerable extent. A reduction in β-adrenergic receptors and an increment in α-adrenergic receptors lead to absolute depression of the adrenergic responses at the peripheral level in hypothyroidism boosting efferent sympathetic activity reaching almost every tissue [5]. As soon as the eccrine glands become edematous, the skin resistance drops, and the conductance augments. It is still unclear whether the modified GSR response is caused by the deposition of myxo edematous fluid over the skin, alteration of sympathetic sudomotor activity by thyroxine, catecholamine concentrations, or widespread immunological impact on the autonomic system [4].

The handgrip strength (HGS) test is another investigation for assessing sympathetic functioning in hypothyroid patients. As thyroid hormone plays a significant role in the myelination of somatic nerves, its deficiency in hypothyroid patients causes disruption in the myelination process leading to a delay in the conduction of impulses for carrying out work. Significantly high diastolic blood pressure has been reported in hypothyroid patients as it depends primarily on vascular resistance that reflects sympathetic reactivity [5].

Skeletal muscle is a common target of hypothyroidism because it requires the binding of T3 to thyroid hormone nuclear receptors for normal muscle development, homeostasis, and regeneration. Hypothyroidism is complicated by neuromuscular symptoms (slowness of movements and tiredness) and signs (easy fatigability and cramps) [6]. Typical hallmarks of this condition are myalgia and mono or polyneuropathy. Not only the pathogenesis of neuromuscular symptoms is largely debated but very little is known about the interrelation between GSR and the autonomic function correlates of hypothyroidism. Anthropometry involves the systematic measurement of the physical properties of the human body, primarily dimensional descriptors of body size and shape [7]. Hypothyroidism patients typically have elevated levels of epicardial adipose tissue.

With the reduction in metabolic rate, a greater association can be predicted between body fat percentage (BFP) and the risk factors further predisposing patients to cardiovascular diseases [8]. Thyroid hormones principally help in maintaining basal metabolic rate which is distorted in hypothyroidism causing decreased thermogenesis and fat metabolism and ultimately leading to an increase in body weight and fat content. This hypothesis is supported by the fact that 15-30% of hypothyroid patients are reported to be overweight [9,10].

Numerous studies have been conducted to determine the effect of BFP due to hypothyroidism while only a few studies have been conducted on HGS and GSR nationally as well as globally [8-10]. According to a study by Mulic et al., patients with subclinical hypothyroidism had higher mean values and statistically significantly higher incidences of elevated values of body mass index (BMI), BFP, waist circumference (WC), and WC/height ratio compared to the control group of euthyroid patients [11]. Similarly, Azevêdo et al. also reported a higher BFP in women diagnosed with hypothyroidism above 35 years of age when compared to those who did not have the same pathology [8]. Bakiner et al. and Lloyd et al. also concluded that the irregularities in thyroid hormone secretion are linked to diseases in lipid metabolism; hence, hypothyroidism patients typically exhibit raised BFP, followed by an alteration in weight [9,12].

A previous study by Dolu et al. demonstrated reduced skin conductance and delayed-onset latencies of GSR in hypothyroid patients [13]. Reduced sympathetic drive and an increase in vagal activity were indicated in hypothyroidism [14], but contrasting results were obtained in an Indian study by Jayakrishnan et al., reflecting an increased sympathetic drive in both hypothyroidism and hyperthyroidism [10].

Comparable results have been reported in subclinical and overt hypothyroid by Syamsunder et al., who reported an increased sympathetic and decreased parasympathetic activity leading to a sympathovagal imbalance in these patients, which was more intense in overt hypothyroid patients. This indicated that the autonomic imbalance could be proportionate with the degree of thyroid hormone deficiency [15]. Additional findings of inverse correlation with serum creatine phosphokinase (CPK) levels suggested that a myopathy state is present in hypothyroid patients, which is supported by a study conducted by Khaleeli et al. [16]. Despite receiving replacement therapy for 9-11 months, half of the patients experienced muscular atrophy. Conversely, clinical follow-up studies by Lankhaar et al. [17] and Duyff et al. [18] revealed a striking improvement in symptoms and a slight improvement in the HGS test, both of which are indicative of improved physical performance.

Despite extensive literature search, there appears to be a gap in the knowledge regarding autonomic function in hypothyroid patients, particularly from this part of the world in terms of HGS and GSR. Because the ANS is primarily involved in energy metabolism and the regulation of the cardiovascular system and because there is a scarcity of scientific documentation on autonomic function in hypothyroid patients, it was thought conceivable to investigate the primary determinants of sympathetic activity, namely, GSR and HGS, to assess the alteration in the ANS in hypothyroidism patients. Additionally, considering the resulting hypothyroidism-related alterations in body composition, this study aims to compare the anthropometric characteristics of hypothyroid-diagnosed females with healthy female controls in a rural Indian setting.

## Materials and methods

Study design and setting

This was a case-control study. The Strengthening the Reporting of Observational Studies in Epidemiology (STROBE) guidelines for case-control studies were used for reporting and preparing the manuscript. The study duration was two months. The study was performed in the Department of Physiology in association with the Department of Anatomy of a rural medical college in central India.

Study population and selection criteria

Study subjects were divided into case and control groups. The sample size was estimated using OpenEpi 3.01 statistical software (Centers for Disease Control and Prevention, Atlanta) assuming a confidence level of 95%, study power of 80%, and a case-to-control ratio of one. The calculated sample size was 23 controls and 23 cases. However, a sample size of 40 controls and 40 cases was considered to accommodate non-responsive subjects.

Diagnosed cases of hypothyroidism aged between 18 and 50 years were referred by consultant physicians from the Department of Medicine. The study was limited to females because they account for the majority of hypothyroid patients. Age-matched euthyroid females who provided consent were recruited as controls.

Subjects suffering from or having a history of co-morbidities such as coronary heart disease; any kind of electrolyte imbalance; diabetes mellitus; hypertension; respiratory, hepatic, renal, neurological, or psychiatric illnesses; endocrine disorders; any hormonal therapy; and those taking drugs affecting autonomic function were excluded from the study.

Consent was obtained or waived by all study participants. Institutional Ethics Committee for Research on Human Subjects of Mahatma Gandhi Institute of Medical Sciences, Wardha issued study approval (approval number: MGIMS/IEC/ANAT/163/2019).

Data sources and measurement of variables

Anthropometric Variables

The age of the subjects was noted in years. The standing height was recorded in centimeters (cm) while they were barefoot with their heels together. Weight in kilograms (kg) was measured in a standing position with light clothes and without footwear. WC was determined using a measuring tape over bare skin or light undergarments. Measurement sites were obtained with the subject assuming a standing position. WC was measured halfway between the lower border of the ribs and the iliac crest in the horizontal plane. Two measurements to the nearest 0.5 cm were recorded. If the variation between the measurements was >2 cm, a third measurement was taken. The mean of the two closest measurements was calculated [19]. BMI was recorded using the Phoenix Height Weight Body Mass Index Machine (Model: PBMI-200, India).

BFP was calculated from the age and WC using the formula given by Lean et al. for the female population. It yielded a more robust prediction of the BFP with the least bias compared to other equations derived by regression analysis [20].

$BFP = (0.439 &times; WC ) + (0.221 &times; Age) - 9.4$

Galvanic Skin Response

GSR recordings were obtained using Power Lab with accompanying equipment and software from AD Instruments (Bella Vista, New South Wales, Australia). As the foundation of the data acquisition solution, the electrical conductance of the skin was recorded and analyzed against a broad range of physiological or psychological signals in real time. By integrating multiple data streams with the LabChart software, powerful connections can be discovered between skin conductance response and physical or emotional stimuli. As GSR measurements operate by detecting electrical (ionic) activity resulting from changes in sweat gland activity, the electrodes used should be sensitive to such changes.

The Equivital wireless physiological monitoring system records a wide range of physiological data via a compact and unobtrusive sensor belt. The GSR sensor connects to the expansion port on the Equivital sensory electronic module (SEM) to enable the recording of GSR signals. It has short leads terminating in standard snap lead connectors suitable for use with disposable electrodes.

The tonic base level driver, whose fluctuations range from seconds to minutes, and the faster-varying phasic component, whose fluctuations range from seconds to minutes, combine to create the GSR signal. Phasic activity fluctuations appear as a steep ascent to an identifiable peak and a gradual descent with respect to the baseline. An event-related skin conductance response, also known as a GSR peak, is a significant change in GSR activity in response to a stimulus. Because skin conductance is modulated by sympathetic activity, it offers direct insights into autonomous regulation.

To record the skin response subjects were investigated after obtaining a history of overnight fasting, any form of exercise in the last 12 hours, and consumption of caffeine in the past eight hours. None of the subjects involved in this study were taking any medication for at least two weeks before testing. The GSR testing apparatus was connected to the vest with a wireless SEM and tied on the wrist of either hand. The data were transmitted wirelessly to a computer system and were acquired with sampling rates of 1-10 Hz and measured in units of micro-Siemens (μS). After the subject had washed, rinsed, and dried their hands, the electrodes were applied to the index and ring fingers with no other electronic devices in close proximity. Because the response depends on body temperature, skin temperature was measured, and if it was under 32°C, the limbs were warmed. The system was linked to an audio file with a sound of high intensity of two-second duration. At first, there was a brief two-minute no-stimuli period to establish the baseline reading. The stimulus was applied twice at an interval of 30 seconds. The difference between the baseline and the maximum value was obtained for each peak value after the application of stimulus which was expressed in micro-Siemens (µS).

Hand Grip Strength

The Grip Force Transducer (MLT004/ST, AD Instruments, Australia) was used to assess the HGS. One value of the maximum voluntary contraction was recorded, which corresponded to 100% of HGS. A set of values collected over a period of 0-2 minutes with intervals of 20 seconds each were used to calculate the mean HGS. Before and after the activity, systolic and diastolic blood pressure measurements, as well as heart rate, were obtained. LabChart software was used to analyze and convert the raw data into Newton (N), which was obtained via the Power Lab data recording equipment [21].

Statistical analysis

Statistical analysis comprised comparison using the Student’s t-test of continuous variables and the chi-Square test for the comparison of categorical (dichotomous) variables. The mean and standard deviation of all studied continuous variables in the normal distribution were done using SPSS version 20.0 (SPSS Inc., Chicago, IL, USA). Linear regression analyses were performed using standard techniques. Pearson’s correlation test was used for examining the correlation between variables. The correlation coefficient (r) was analyzed for statistical significance. A p-value less than 0.05 was considered statistically significant as per two-tailed tests.

## Results

This study included 40 female hypothyroid patients aged 18-50 years as cases and 40 age-matched healthy subjects enrolled as controls. Cases with hypothyroidism had statistically significantly elevated mean thyroid-stimulating hormone (TSH) values (9.70 ± 4.73 mIU/mL) compared to the TSH levels of controls. The mean age of cases and controls was 30.14 ± 5.91 years and 29.37 ± 6.59 years, respectively. Two-tailed Student’s t-test results of the basal levels of thyroid hormones, namely, free tri-iodothyronine (FT3), and free thyroxine (FT4), showed an overall significant difference between cases and controls revealing lower serum thyroid hormone levels than those of healthy subjects. The descriptive statistics of the anthropometric and clinical parameters of the two study groups are depicted in Table 1.

**Table 1:** Descriptive statistics of anthropometric and clinical parameters of the study groups. *: All reported p-values are based on Student’s t-test applied between the difference in the means of the control and case groups. S: significant; NS: non-significant; SD: standard deviation; BMI: body mass index; BFP: body fat percentage; BP: blood pressure

Parameters	Cases (n = 40), mean ± SD	Controls (n = 40), mean ± SD	P-value*
Age (years)	30.14 ± 5.91	29.37 ± 6.59	0.584 (NS)
Height (cm)	157.57 ± 6.25	158.33 ± 4.26	0.527 (NS)
Weight (kg)	55.72 ± 10.82	58.54 ± 11.89	0.271 (NS)
Waist circumference (cm)	85.81 ± 10.39	80.90 ± 11.18	0.045 (S)
Heart rate (beats/minute)	79.18 ± 9.21	77.5 ± 4.39	0.300 (NS)
BMI (kg/m^2^)	23.88 ± 4.04	23.35 ± 4.66	0.588 (NS)
BFP (%)	35.38 ± 6.74	31.72 ± 5.63	0.010 (S)
Systolic BP (mmHg)	115.95 ± 18.16	111.79 ± 6.23	0.174 (NS)
Diastolic BP (mmHg)	75.74 ± 11.95	73.42 ± 6.79	0.289 (NS)

From Table 1, it is evident that compared to the control group, cases with hypothyroidism had significantly higher values of WC (p = 0.045) and BFP (p = 0.010). There was no statistically significant difference between the two groups regarding age, weight, height, heart rate, and systolic and diastolic blood pressure.

GSR indices of females of both study groups are expressed as mean ± standard deviation in Table 2. A statistically significant difference was noted between the GSR amplitudes of the two study groups (p = 0.002).

**Table 2:** GSR indices between the study groups. *: All reported p-values are based on Student’s t-test applied between the difference in the means of the control and case groups. S: significant; NS: non-significant; SD: standard deviation; GSR: galvanic skin response; μS: micro-Seimens

Parameters	Cases (n = 40), mean ± SD	Controls (n = 40), mean ± SD	P-value*
GSR baseline (μS)	4.19 ± 1.62	3.61 ± 2.39	0.208 (NS)
GSR peak value (μS)	5.27 ± 2.32	5.51 ± 3.52	0.720 (NS)
GSR amplitude (μS)	1.34 ± 1.14	2.40 ± 1.86	0.002 (S)

The differences in the HGS indices when compared among the two groups were not statistically significant, as depicted in Table 3.

**Table 3:** Hand grip strength indices between the study groups. *: All reported p-values are based on Student’s t-test applied between the difference in the means of the control and case groups. S: significant; NS: non-significant; SD: standard deviation; BP: blood pressure

Parameters	Cases (n = 40), mean ± SD	Controls (n = 40), mean ± SD	P-value*
Heart rate (beats/minute)	79.18 ± 9.21	77.5 ± 4.39	0.166 (NS)
Maximum voluntary contraction (N)	201 ± 60	204 ± 41	0.795 (NS)
Mean handgrip strength (N)	51.51 ± 15.06	52.8 ± 10.8	0.651 (NS)
Systolic BP before exercise (mmHg)	111.8 ± 6.2	116.0 ± 18.2	0.174 (NS)
Diastolic BP before exercise (mmHg)	73.4 ± 6.8	75.7 ± 12.0	0.289 (NS)
Systolic BP after exercise (mmHg)	117.2 ± 13.7	114.8 ± 5.9	0.311 (NS)
Diastolic BP after exercise (mmHg)	76.1 ± 11.1	77.1 ± 6.8	0.631 (NS)

**Figure 1:**
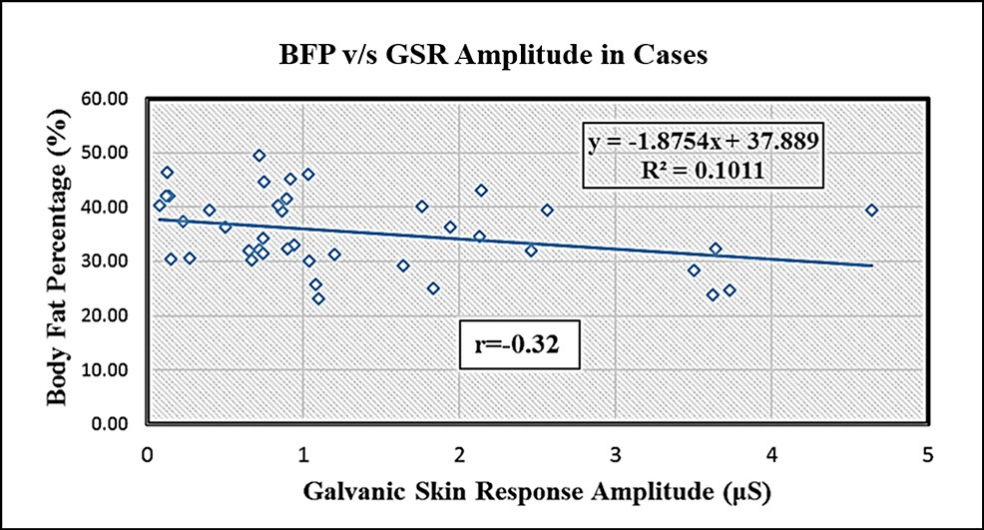
Scatter plot of body fat percentage versus galvanic skin response amplitude in cases.

Table 4 shows a correlation between BFP and GSR. There was a statistically significant negative correlation (r = -0.32, p = 0.038) indicating a reduction in the GSR amplitude with a corresponding increase in the BFP value. This is depicted in Figure 1 as a scatter plot.

**Table 4:** Correlation of BFP with GSR between case and control groups. S: significant; NS: non-significant; GSR: galvanic skin response; BFP: body fat percentage

Study group	BFP	GSR amplitude	r value	P-value
Cases	35.38 ± 6.74	1.34 ± 1.14	-0.32	0.038 (S)
Controls	31.72 ± 5.63	2.40 ± 1.86	-0.09	0.669 (NS)

**Figure 2:**
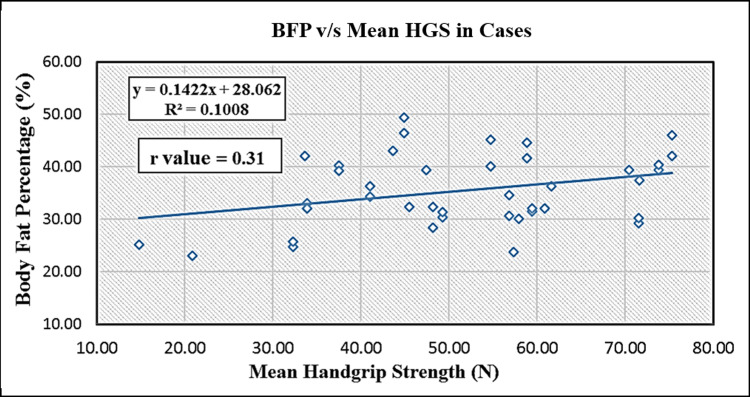
Scatter plot of body fat percentage versus mean hand grip strength in cases.

As shown in Table 5, BFP and mean HGS measured by a grip force transducer in female hypothyroid cases, a positive correlation (r = 0.31, p = 0.046) was achieved, which was statistically significant. The same is illustrated in Figure 2 as a scatter plot.

**Table 5:** Correlation of BFP with the mean handgrip strength between the case and control groups. S: significant; NS: non-significant; BFP: body fat percentage

Study group	BFP	Mean handgrip strength (N)	r value	P-value
Cases	35.38 ± 6.74	51.51 ± 15.06	0.31	0.046 (S)
Controls	31.72 ± 5.63	52.8 ± 10.8	-0.04	0.801 (NS)

## Discussion

Numerous studies have shown that autonomic dysfunction occurs in several systemic diseases [10,15]. In particular, the clinical presentation of hypothyroidism suggests deficient adrenergic stimulation. In this study, using a combination of tests that were not previously employed in thyroid patients, the autonomic function was evaluated in hypothyroid patients and compared with healthy normal controls.

This study examined the basal levels of GSR in response to an acoustic stimulus in both hypothyroid and healthy subjects. On comparison of mean GSR amplitude in both groups in our study, we found a statistically significant difference (p = 0.002), which is not in agreement with previous studies [4,5]. The abnormal GSR obtained in our subjects could be a result of altered hypothalamic-pituitary-thyroid axis activity. The shift in GSR may be caused by the substantial changes in thyroid status that occur in the metabolism of several biogenic amines in the brain [5]. The transfer of ions across the cell membrane of the skin sweat glands has previously been attributed to the diminished GSR, which likely represents sudomotor activity [22]. Shahani et al. [23] have quantified this phenomenon in the form of a sympathetic skin response linked with the activity of unmyelinated post-ganglionic sympathetic fibers. Although the mechanisms underlying the relationship between GSR and the levels of thyroid hormones remain unknown, the GSR reflects several factors, for example, the structure and hydration of the epidermis, electrical membrane effects and anatomy, and degree of eccrine sweat gland activity, epidermal thickness, and changes in cell replication [24].

Because hypothyroidism is known to cause an increase in BFP, it results in the accumulation of subcutaneous mucopolysaccharides. To clarify the ambiguity regarding the precise nature of the relationship between thyroid hormones and autonomic nerve activity our findings clearly depict a negative correlation between BFP and GSR, which could be explained by the shift in the balance of alpha and beta receptors in favor of the alpha receptors which has also been postulated in previous studies [4,5].

Evidence supporting the idea that hypothyroidism will likely have long-term negative implications and is expected to cause the suppressive actions of thyroid hormone on the muscular systems resulting in reduced HGS is well portrayed by our findings. Our observation of reduced muscle grip is in concordance with that of Gallo et al. [6], who also demonstrated a clear trend of reduced strength in their hypothyroid patients. As skeletal muscle is a prime target organ for thyroid hormones, this does not seem unlikely. The rapid decline in energy reserves of exercising hypothyroid muscle has been previously attributed to a reduction in mitochondrial activity [25]. The presence of T3 receptors on the mitochondrial membrane of skeletal muscle also confirms a direct impact of thyroid hormones on oxidative metabolism and may provide a biochemical basis for the muscle dysfunction observed in hypothyroidism. There is a myopathy state in hypothyroid subjects which was already implicated by the finding of inverse correlation of HGS with serum CPK levels [6]. It has also been corroborated by a study reported by Khaleeli et al. [16].

In underweight, normal-weight, and overweight adolescents, Lad et al. recorded and measured the BFP, HGS, and handgrip endurance. They postulated a complex correlation between these parameters. Lad et al. [26] concluded that the augment in BFP may reduce the handgrip endurance but not the HGS. Both the underweight and normal-weight males and the overweight females showed a significantly positive correlation between BFP and HGS [26].

Our results of statistically significant positive linear correlation (r = 0.31, p = 0.046) obtained when the relationship between mean BFP and mean HGS values of our subjects were analyzed are in line with them and in agreement with the findings of Hulens et al. [27]. This association explains that an increase in the BFP does not have a detrimental effect on overweight and normal-weight females.

This study has some limitations. The fact that there was no gradation between the two groups of participants for the assessment of autonomic status in our study indicates that the limited number of subjects in each group may not have accurately depicted the true nature of thyroid dysfunction.

## Conclusions

The correlations observed between GSR and muscle grip strength with BFP in this study have highlighted the need for anthropometric measurements to be performed before initiating any treatment regime and not just once therapy has begun. They can also have immense diagnostic value when patients are consulting with their treating physician.

In a nutshell, the results of this study indicate that patients with hypothyroidism have significant changes in anthropometric parameters such as WC, BFP, lowered GSR, and reduced muscle grip strength. Timely and precise identification of these parameters in hypothyroid patients opens up the possibilities of specific therapeutic interventions leading to improvement in the responses of the patients.

## References

[1] Almas SP, Werneck FZ, Coelho EF, Teixeira PF, Vaisman M (2017). Heart rate kinetics during exercise in patients with subclinical hypothyroidism. J Appl Physiol (1985).

[2] Unnikrishnan AG, Kalra S, Sahay RK, Bantwal G, John M, Tewari N (2013). Prevalence of hypothyroidism in adults: an epidemiological study in eight cities of India. Indian J Endocrinol Metab.

[3] Deshmukh V, Behl A, Iyer V, Joshi H, Dholye JP, Varthakavi PK (2013). Prevalence, clinical and biochemical profile of subclinical hypothyroidism in normal population in Mumbai. Indian J Endocrinol Metab.

[4] Nagai Y, Jones CI, Sen A (2019). Galvanic skin response (GSR)/electrodermal/skin conductance biofeedback on epilepsy: a systematic review and meta-analysis. Front Neurol.

[5] Gautam S, Tandon OP, Awashi R, Sekhri T, Sircar SS (2003). Correlation of autonomic indices with thyroid status. Indian J Physiol Pharmacol.

[6] Gallo D, Piantanida E, Veronesi G (2017). Physical performance in newly diagnosed hypothyroidism: a pilot study. J Endocrinol Invest.

[7] Islam A, Asadujjaman M, Nuruzzaman M, Hossain M (2013). Ergonomics consideration for hospital bed design: a case study in Bangladesh. J Modern Sci Technol.

[8] Azevêdo LM, Oliveira MF, Dos Santos M (2018). Anthropometric profile of sedentary women with and without hypothyroidism. Rev Andal Med Deporte.

[9] Bakiner O, Bozkirli E, Ersozlu Bozkirli ED, Ozsahin K (2013). Correction of hypothyroidism seems to have no effect on body fat. Int J Endocrinol.

[10] Jayakrishnan G, Pal GK, Kamalanathan S, Pal P, Sirisha A, Nanda N (2017). Association of prehypertension status with cardiovascular risks in subclinical hypothyroidism. Int J Clin Exp Physiol.

[11] Mulic M, Muminovic S, Skrijelj F, Mulic M, Vujosevic S (2018). The importance of anthropometric paremeters in patients with subclinical hypothyroidism. Sanamed.

[12] de Lloyd A, Bursell J, Gregory JW, Rees DA, Ludgate M (2010). TSH receptor activation and body composition. J Endocrinol.

[13] Dolu N, Süer C, Ozesmi C, Keleştimur F, Ozcan Y (1999). Electrodermal activity in hypothyroid patients and healthy subjects. Thyroid.

[14] Xing H, Shen Y, Chen H, Wang Y, Shen W (2001). Heart rate variability and its response to thyroxine replacement therapy in patients with hypothyroidism. Chin Med J (Engl).

[15] Syamsunder AN, Pal P, Kamalanathan CS (2014). Dyslipidemia and low‑grade inflammation are associated with sympathovagal imbalance and cardiovascular risks in subclinical and overt hypothyroidism. Int J Clin Exp Physiol.

[16] Khaleeli AA, Gohil K, McPhail G, Round JM, Edwards RH (1983). Muscle morphology and metabolism in hypothyroid myopathy: effects of treatment. J Clin Pathol.

[17] Lankhaar JA, de Vries WR, Jansen JA, Zelissen PM, Backx FJ (2014). Impact of overt and subclinical hypothyroidism on exercise tolerance: a systematic review. Res Q Exerc Sport.

[18] Duyff RF, Van den Bosch J, Laman DM, van Loon BJ, Linssen WH (2000). Neuromuscular findings in thyroid dysfunction: a prospective clinical and electrodiagnostic study. J Neurol Neurosurg Psychiatry.

[19] (2000). Obesity: preventing and managing the global epidemic. Report of a WHO consultation. World Health Organ Tech Rep Ser.

[20] Lean ME, Han TS, Deurenberg P (1996). Predicting body composition by densitometry from simple anthropometric measurements. Am J Clin Nutr.

[21] Sushmitha S, Kothari R, Mittal G (2023). Exploring the relationship between the indices of body composition with grip strength performance and peak VO2. Cureus.

[22] Yokota T, Matsunaga T, Okiyama R, Hirose K, Tanabe H, Furukawa T, Tsukagoshi H (1991). Sympathetic skin response in patients with multiple sclerosis compared with patients with spinal cord transection and normal controls. Brain.

[23] Shahani BT, Halperin JJ, Boulu P, Cohen J (1984). Sympathetic skin response--a method of assessing unmyelinated axon dysfunction in peripheral neuropathies. J Neurol Neurosurg Psychiatry.

[24] Thorell LH, Kjellman BF, d'Elia G (1993). Electrodermal activity in relation to basal and postdexamethasone levels of thyroid stimulating hormone and basal levels of thyroid hormones in major depressive patients and healthy subjects. Psychiatry Res.

[25] Kaminsky P, Robin-Lherbier B, Brunotte F (1992). Energetic metabolism in hypothyroid skeletal muscle, as studied by phosphorus magnetic resonance spectroscopy. J Clin Endocrinol Metab.

[26] Lad UP, Satyanarayana P, Shisode-Lad S, Siri ChC, Kumari NR (2013). A study on the correlation between the body mass index (BMI), the body fat percentage, the handgrip strength and the handgrip endurance in underweight, normal weight and overweight adolescents. J Clin Diagn Res.

[27] Hulens M, Vansant G, Lysens R, Claessens AL, Muls E, Brumagne S (2001). Study of differences in peripheral muscle strength of lean versus obese women: an allometric approach. Int J Obes Relat Metab Disord.

